# Diagnostic accuracy of rapid integrated molecular testing for major respiratory viral infections: a systematic review and meta-analysis

**DOI:** 10.3389/fmed.2026.1909562

**Published:** 2026-09-11

**Authors:** Chenzi Liu, Daojun Yu

**Affiliations:** 1 Department of Laboratory Medicine, The Second Affiliated Hospital of Zhejiang Chinese Medical University, Hangzhou, China; 2 Department of Laboratory Medicine, The Fourth School of Clinical Medicine, Zhejiang Chinese Medical University, Hangzhou First People's Hospital, Hangzhou, China

**Keywords:** diagnostic accuracy, meta-analysis, point-of-care testing, rapid integrated molecular testing, respiratory viruses, sensitivity, specificity

## Abstract

**Background:**

Rapid integrated molecular tests are automated sample-to-answer nucleic acid amplification systems that provide respiratory virus results within approximately 120 min. Because their use ranges from genuine near-patient sites to rapid laboratory workflows, testing environments must be clearly classified.

**Methods:**

PubMed, Embase, and the Cochrane Library were searched from inception through June 2026 without language restrictions. Diagnostic-accuracy studies evaluating integrated rapid molecular assays for influenza A, influenza B, respiratory syncytial virus (RSV), or SARS-CoV-2 were eligible. For the primary analysis, one prespecified platform and one non-nested cohort were selected from each study, and target-specific 2 × 2 counts were aggregated into one study-level composite record. Sensitivity and specificity were jointly synthesized using a bivariate random-effects model. The overall analysis was considered exploratory, with pathogen-specific findings prioritized. Pathogen- and setting-specific subgroup analyses, leave-one-study-out analyses, QUADAS-2 assessment, and Deeks' funnel-plot asymmetry testing were performed.

**Results:**

Of 1,337 identified records, 43 underwent full-text assessment and 23 were excluded. Twenty studies contributed 20 independent study-level records. Sensitivity and specificity were 0.970 and 0.987 for influenza A, 0.961 and 0.992 for influenza B, 0.955 and 0.992 for RSV, and 0.950 and 0.992 for SARS-CoV-2, respectively. The exploratory overall analysis yielded a sensitivity of 0.971 [95% confidence interval (CI), 0.955–0.982], specificity of 0.991 (95% CI, 0.980–0.996), and HSROC area under the curve of 0.957. Five studies were conducted in genuine near-patient settings and 15 in laboratory settings. Leave-one-study-out estimates ranged from 0.968 to 0.974 for sensitivity and 0.989 to 0.992 for specificity. Deeks' test showed no statistically significant funnel-plot asymmetry (*P* = 0.269).

**Conclusions:**

Pathogen-specific analyses indicated high diagnostic accuracy for rapid integrated molecular testing of influenza A, influenza B, RSV, and SARS-CoV-2. Because platforms, specimens, populations, settings, designs, and reference methods varied, the overall estimates and HSROC area should be interpreted only as exploratory summaries. Most studies were laboratory based, and direct clinical or economic benefits were not established.

## 1 Introduction

Respiratory viral infections impose a substantial burden on patients and healthcare systems worldwide (1). Influenza viruses, respiratory syncytial virus (RSV), and severe acute respiratory syndrome coronavirus 2 (SARS-CoV-2) are major causes of outpatient consultations, emergency department visits, hospital admissions, and preventable deaths (2). The risk of severe disease is particularly high among young children, older adults, pregnant women, and immunocompromised individuals (3). The coronavirus disease 2019 pandemic further highlighted the importance of timely pathogen identification for clinical management, infection prevention, surveillance, and outbreak control (4).

Accurate etiological diagnosis based on clinical presentation alone is difficult because respiratory viral infections commonly produce overlapping symptoms, including fever, cough, rhinorrhea, sore throat, and dyspnea. Viral culture has historically been used as a reference method, but its long turnaround time and requirement for specialized laboratory facilities limit its routine clinical application (5). Rapid antigen tests provide results quickly but generally have lower sensitivity than nucleic acid amplification tests, particularly in specimens with low viral concentrations (6). Laboratory-based reverse-transcription polymerase chain reaction offers high analytical sensitivity and specificity but may involve centralized testing, batch processing, specimen transport, and delayed reporting (7). Consequently, a diagnostic approach that combines molecular accuracy with a shorter and simpler testing workflow is clinically desirable.

Rapid integrated molecular tests are automated or near-automated nucleic acid amplification systems that combine several analytical steps within a sample-to-answer workflow and typically provide results within approximately 120 min. Representative platforms include cobas Liat, Xpert Xpress, ID NOW, BioFire FilmArray and SPOTFIRE, Luminex ARIES, Idylla, and STANDARD M10. Some of these systems are implemented at or near the point of patient care, whereas others are evaluated or operated in local, satellite, or central laboratories. Therefore, rapid analytical turnaround and integrated automation should not automatically be regarded as equivalent to genuine point-of-care implementation. Explicit classification according to testing location and workflow is necessary when evaluating their diagnostic performance and potential clinical application.

Rapid availability of multiplex PCR results may shorten the time to pathogen identification and support earlier clinical decision-making (8) Integrated molecular platforms offer several potential advantages. Closed or near-closed sample-to-answer workflows can reduce manual processing, while multiplex assays can detect multiple respiratory pathogens from a single specimen. In a multicenter evaluation, the ePlex Respiratory Pathogen Panel provided simultaneous detection of 19 viral and two bacterial targets and demonstrated high agreement with the comparator respiratory panel (9) Platform-specific evaluations have also demonstrated favorable analytical performance; for example, the Xpert Xpress SARS-CoV-2/Flu/RSV assay showed high analytical sensitivity and specificity for its intended viral targets (10).

Previous diagnostic evaluations have reported favorable performance for individual platforms, but the evidence is heterogeneous with respect to the target pathogen, assay platform, specimen type, patient population, reference standard, and testing setting. In addition, individual studies frequently report multiple pathogen-, platform-, specimen-, or setting-specific datasets. Treating all such datasets as statistically independent may give disproportionate weight to studies contributing multiple results and may produce overly precise summary estimates. A synthesis based on independent study-level records is therefore required.

Accordingly, this systematic review and meta-analysis aimed to evaluate the diagnostic accuracy of rapid integrated molecular testing for influenza A, influenza B, RSV, and SARS-CoV-2. The study-level overall synthesis was retained to provide a broad description of the performance of rapid, integrated sample-to-answer molecular systems as a class and to permit comparison with earlier broad reviews. It was not intended to imply that different pathogens, platforms, specimen types, populations, or reference methods are clinically interchangeable. The primary analysis used one prespecified study-level composite record per included study to preserve the study as the unit of independence, whereas pathogen-specific analyses were given greater weight in clinical interpretation. We also distinguished genuine near-patient implementation from laboratory-evaluated rapid integrated testing. The review focused on diagnostic accuracy and did not directly evaluate the effects of testing on treatment decisions, healthcare utilization, infection-control outcomes, costs, or patient outcomes.

## 2 Methods

### 2.1 Study design and reporting guideline

This systematic review and meta-analysis of diagnostic test accuracy was conducted and reported in accordance with the Preferred Reporting Items for Systematic Reviews and Meta-Analyses 2020 statement (PRISMA 2020) and the PRISMA extension for Diagnostic Test Accuracy studies (PRISMA-DTA) (11).

### 2.2 Literature search strategy

PubMed, Embase, and the Cochrane Library were searched from database inception through June, 2026. No language restrictions were applied. The search strategies combined controlled vocabulary, including Medical Subject Headings and Emtree terms, with free-text terms related to rapid integrated molecular testing, point-of-care testing, and respiratory viral infections. The complete database-specific search strategies and the numbers of records retrieved from each database are provided in Supplementary File S1. The reference lists of eligible reports and relevant systematic reviews were also screened manually to identify additional studies.

### 2.3 Eligibility criteria

For this review, rapid integrated molecular tests were defined as automated or near-automated nucleic acid amplification systems that used an integrated sample-to-answer workflow, required minimal manual processing, and provided results within 120 min. Eligible tests were classified into two prespecified operational categories: genuine near-patient rapid integrated testing and laboratory-evaluated rapid integrated testing. Genuine near-patient testing referred to assays performed in a clinic, emergency department, hospital ward, bedside area, CLIA-waived site, or comparable decentralized setting. Laboratory-evaluated rapid integrated testing referred to rapid sample-to-answer systems evaluated in local, satellite, or central laboratory settings. Laboratory-evaluated systems were included in the overall analysis but were not classified as genuine near-patient point-of-care tests. Detailed operational definitions are provided in Supplementary Table S1.

Studies were eligible if they: (i) evaluated an integrated or near-integrated nucleic acid amplification assay for influenza A, influenza B, respiratory syncytial virus (RSV), or SARS-CoV-2; (ii) used human respiratory specimens; (iii) compared the index test with an acceptable molecular reference standard; and (iv) reported sufficient data to reconstruct a complete (2 \times 2) contingency table comprising true-positive, false-positive, false-negative, and true-negative results.

Reviews, editorials, letters without original data, conference abstracts, guidelines, protocols, case reports, and nonclinical analytical studies were excluded. Studies were also excluded if they evaluated nonrespiratory targets, used only conventional centralized or batch molecular workflows, did not use an acceptable molecular reference standard, or did not provide sufficient data to reconstruct a complete (2 \times 2) table. When multiple reports included overlapping participants or specimens, the most complete report was retained.

### 2.4 Study selection

All retrieved records were imported into NoteExpress, and duplicate records were removed. Two authors independently screened titles and abstracts and subsequently assessed the full texts of potentially eligible reports. Disagreements at either stage were resolved through discussion and consensus.

### 2.5 Data extraction

Two authors independently extracted the following information using a standardized data-extraction form: first author, publication year, country, study design, patient population, clinical setting, age group, index-test platform, operational testing category, target pathogen, assay target gene(s) when reported, specimen type, exact reference method, positivity criterion or manufacturer-defined interpretation rule, discordant-result resolution procedure, sample size, prevalence, and the numbers of true-positive, false-positive, false-negative, and true-negative results. Disagreements were resolved through discussion and consensus.

Individual reports could provide multiple pathogen-, platform-, specimen-, setting-, or cohort-specific (2 \times 2) datasets. These datasets were linked using a common report identifier and were not treated as statistically independent. For the primary analysis, one platform and one non-nested clinical cohort were selected from each report according to a prespecified hierarchy: genuine near-patient implementation, prospective evaluation of clinical specimens, the largest eligible cohort, and the most completely reported molecular reference standard.

Within the selected platform and cohort, target-specific (2 \times 2) counts were aggregated to construct one report-level composite target-test record. Consequently, the resulting denominators represented target-test pairs rather than unique patients or specimens. For pathogen-specific subgroup analyses, no more than one selected record per report was included for each pathogen.

### 2.6 Quality assessment

The methodological quality and applicability of the included studies were independently assessed by two authors using the Quality Assessment of Diagnostic Accuracy Studies 2 instrument (QUADAS-2) developed by Whiting et al. (12). Risk of bias was evaluated across four domains: patient selection, index test, reference standard, and flow and timing. Applicability concerns were evaluated for patient selection, the index test, and the reference standard. Each domain was judged as having low, high, or unclear risk or concern. Disagreements were resolved through discussion and consensus.

### 2.7 Statistical analysis

All statistical analyses were performed using R version 4.4.3. The primary analysis included one independent report-level composite target-test record per eligible report. The overall synthesis was retained only to provide a broad exploratory description of rapid integrated sample-to-answer molecular systems as a test class and to permit comparison with earlier broad reviews. It was not intended to provide a universally transferable estimate for any individual pathogen, assay platform, specimen type, patient population, testing setting, study design, or reference method. Sensitivity and specificity were jointly synthesized using a bivariate random-effects model fitted on the logit scale. Summary sensitivity and specificity were reported with 95% confidence intervals. The positive likelihood ratio, negative likelihood ratio, and diagnostic odds ratio were derived from the summary sensitivity and specificity estimates.

Forest plots were generated to display study-level sensitivity and specificity with their corresponding 95% confidence intervals. A hierarchical summary receiver operating characteristic curve was constructed to describe the joint distribution of sensitivity and specificity, and the corresponding area under the curve was calculated.

Prespecified subgroup analyses were performed according to target pathogen and operational testing setting. Pathogen-specific analyses included influenza A, influenza B, RSV, and SARS-CoV-2. Testing-setting analyses compared genuine near-patient rapid integrated testing with laboratory-evaluated rapid integrated testing. These subgroup comparisons were considered exploratory because testing setting could be confounded by differences in platform, study design, patient population, and specimen type. Additional subgroup analyses and meta-regression according to assay platform, specimen type, age group, clinical setting, study design, and reference standard were considered but were not performed. The primary analysis included only 20 independent records, and several categories contained only one or two studies. Moreover, assay platform, testing setting, specimen type, patient population, study design, and reference method overlapped substantially. Further stratification would frequently have resulted in only one or two studies per subgroup, whereas multivariable bivariate meta-regression would have been underpowered, statistically unstable, and vulnerable to collinearity and ecological confounding. Such analyses could therefore have produced unreliable or misleading estimates.

The robustness of the primary estimates was examined using a leave-one-report-out sensitivity analysis, in which the bivariate model was refitted after sequentially excluding each report. Small-study effects were assessed using Deeks' funnel-plot asymmetry test. A two-sided (*P)* value of less than 0.10 in Deeks' test was considered indicative of funnel-plot asymmetry. For all other statistical analyses, two-sided (*P*) values of less than 0.05 were considered statistically significant.

## 3 Results

### 3.1 Literature search and study selection

**Figure 1:**
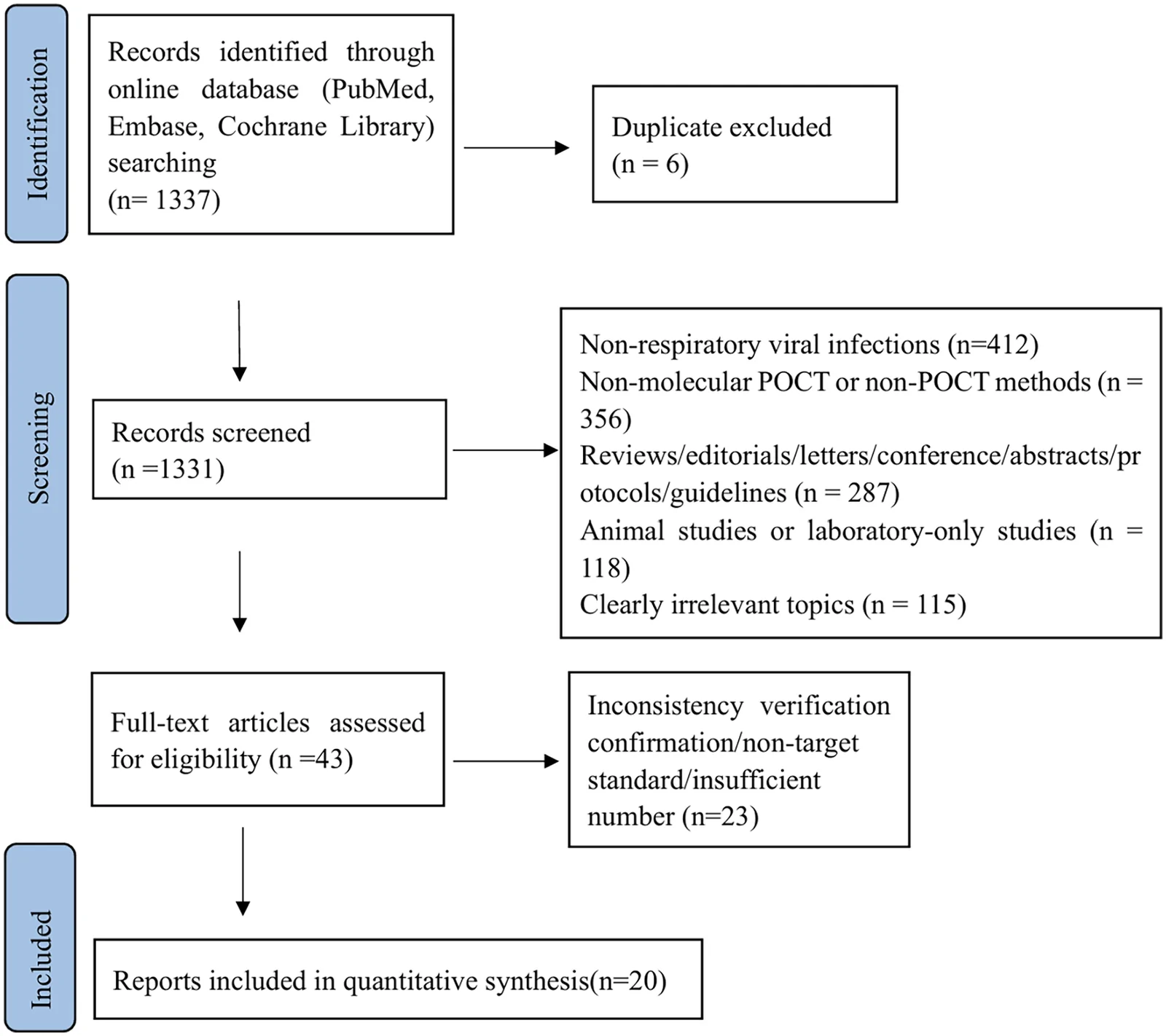
PRISMA flow diagram.

The database searches identified 1,337 records, including 448 from PubMed, 784 from Embase, and 105 from the Cochrane Library. After six duplicate records were removed, 1,331 titles and abstracts were screened. Of these, 43 reports were retrieved and assessed for full-text eligibility. Twenty-three full-text reports were excluded because they did not meet the prespecified eligibility criteria. Ultimately, 20 studies were included in the qualitative and quantitative syntheses, each contributing one independent study-level primary record (Figure 1).

### 3.2 Characteristics of included studies

The 20 included studies were published between 2014 and 2025. Five studies evaluated rapid integrated molecular testing in genuine near-patient settings, whereas 15 evaluated rapid integrated systems in laboratory settings. The represented platform families were GeneXpert/Xpert (seven studies), cobas Liat and ID NOW/Alere i (three studies each), Idylla and BioFire FilmArray/SPOTFIRE (two studies each), and STANDARD M10, Luminex NxTAG, and Enigma MiniLab (one study each). Seventeen studies evaluated PCR-based methods and three evaluated isothermal nucleic acid amplification assays. The study populations comprised pediatric or pediatric/adolescent cohorts in three studies, an adult cohort in one, mixed-age populations in 12, and populations with age not reported in four. Nine studies explicitly used nasopharyngeal swabs, seven reported broader respiratory-specimen categories, and four used other or mixed nasal/nasopharyngeal specimen categories. Reference methods included laboratory RT-PCR, commercial comparator molecular assays, composite molecular reference standards, and viral culture combined with RT-PCR. Study-level information on design, setting, platform, molecular method, target pathogen, specimen type, patient population, age group, and reference standard is provided in Table 1.

**Table 1:** Characteristics of the included studies and selected primary records.

Study	Country	Study design	Testing setting	Selected index test	Molecular method	Target pathogen(s)	Specimen type	Patient population	Age group	Reference standard	TP	FP	FN	TN	Target-test total
Haigh et al. (14)	UK	Prospective service evaluation	Genuine near-patient	GeneXpert local near-patient testing	Cartridge-based multiplex real-time RT-PCR	Influenza A/B, RSV	Respiratory specimens	Hospital patients (ambulatory care, ED, and wards)	/	Laboratory RT-PCR*	111	35	4	1,623	1,773
Chen et al. (15)	China	Diagnostic accuracy study	Laboratory-evaluated	Xpert Xpress Flu/RSV	Automated cartridge-based multiplex real-time RT-PCR	Influenza A/B	NPA/NPS	Symptomatic patients from pediatric/adult wards, ICU, and outpatients	Mixed; 11 month–92 year	Laboratory-developed multiplex RT-PCR*	104	0	1	163	268
Mitamura et al. (16)	Japan	Clinical evaluation	Laboratory-evaluated	ID NOW Influenza A&B 2	Isothermal nucleic acid amplification	Influenza A/B	Nasopharyngeal swabs	Children and adults with influenza-like illness	Mixed; children 1 month–15 year plus adults	Laboratory RT-PCR*	164	0	5	373	542
Jensen et al. (17)	Denmark	Diagnostic comparison	Laboratory-evaluated	Xpert Xpress	Cartridge-based quadruplex RT-PCR	Influenza A/B, RSV, SARS-CoV-2	Respiratory specimens	Patients undergoing routine respiratory-virus testing	Mixed; 12 day–94 year	Comparator molecular assay*	320	3	0	673	996
Wouters et al (18).	Belgium	Retrospective comparison	Laboratory-evaluated	Idylla Respiratory Panel	Fully integrated multiplex real-time RT-PCR	Influenza A/B, RSV	Nasopharyngeal swabs	Hospital patients with influenza-like illness	Mixed; ≤6 to ≥65 year	Comparator molecular assay*	172	7	4	879	1,062
Zafiropoulos et al. (19)	Greece	Diagnostic comparison	Laboratory-evaluated	BioFire Respiratory Panel 2 Plus	Nested multiplex RT-PCR	Influenza A/B combined	Respiratory specimens	Hospitalized patients with acute respiratory infection	Mixed; 15 day–85 year	Alere i Influenza A & B assay*	28	6	0	63	97
Johnson et al. (20)	Canada	Diagnostic accuracy study	Laboratory-evaluated	Xpert Xpress CoV-2/Flu/RSV Plus	Automated cartridge-based multiplex RT-qPCR	Influenza A/B, RSV, SARS-CoV-2	Nasopharyngeal swabs	Patients represented by remnant clinical specimens	/	Comparator molecular assay*	122	0	1	517	640
Banerjee et al. (21)	USA	Comparative study	Laboratory-evaluated	cobas Liat	Automated multiplex real-time RT-PCR	Influenza A/B	Nasopharyngeal swabs	Children represented by archived clinical specimens	Pediatric; 2–80 month	Composite molecular reference*	122	3	1	324	450
Cohen et al. (22)	USA	Multicenter diagnostic accuracy study	Genuine near-patient	Xpert Flu/RSV Xpress	Automated cartridge-based real-time RT-PCR	Influenza A/B, RSV	Nasopharyngeal swabs	Symptomatic ED, urgent-care, outpatient, and inpatient patients	Mixed; ≤5 to ≥60 year	ProFlu + assay*	578	126	5	6,596	7,305
Farfour E et al. (23)	France	Diagnostic comparison	Laboratory-evaluated	Idylla SARS-CoV-2/Flu/RSV	Fully integrated multiplex RT-PCR	Influenza A, RSV, SARS-CoV-2	Respiratory specimens	Symptomatic adults presenting to the ED	Adult	Comparator molecular assay*	47	0	9	598	654
McElvania et al. (24)	USA	Multicenter evaluation	Genuine near-patient	Xpert Xpress CoV-2/Flu/RSV Plus	Automated cartridge-based multiplex RT-PCR	Influenza A/B, RSV, SARS-CoV-2	Nasal/nasopharyngeal swabs	Patients with upper respiratory tract infection symptoms	Mixed; ≤5 to ≥60 year	Comparator molecular assay*	1,114	67	18	13,442	14,641
Smit et al. (13)	Netherlands	Comparative accuracy study	Laboratory-evaluated	STANDARD M10	Cartridge-based multiplex real-time RT-PCR	Influenza A/B, RSV, SARS-CoV-2	Respiratory specimens	ED patients with respiratory symptoms	NR	Comparator molecular testing*	145	0	11	456	612
Chan et al. (25)	China	Clinical diagnostic accuracy study	Laboratory-evaluated	BIOFIRE SPOTFIRE RP	Nested multiplex RT-PCR	Influenza A/B	Nasopharyngeal swabs	Inpatients and outpatients undergoing routine respiratory testing	Mixed; predominantly children	BioFire FilmArray Respiratory Panel*	69	0	0	499	568
Chen et al. (26)	China	Clinical evaluation	Laboratory-evaluated	Luminex NxTAG Respiratory Pathogen Panel	Single-tube multiplex RT-PCR with bead-based hybridization	Influenza A/B, RSV	Nasopharyngeal swabs	Patients with symptomatic respiratory tract infection	/	BioFire FilmArray Respiratory Panel and singleplex real-time PCR	104	0	1	163	268
Wahrenbrock et al. (27)	USA	Method-comparison study	Laboratory-evaluated	Xpert Flu/RSV XC	Automated cartridge-based real-time RT-PCR	Influenza A/B, RSV	Nasopharyngeal swabs	Patients represented by archived clinical specimens	Mixed; 17 month–93 year	FilmArray Respiratory Panel*	110	0	3	274	387
Douthwaite et al. (28)	UK	Diagnostic accuracy study	Genuine near-patient	Enigma MiniLab	Automated fluorogenic RT-PCR	Influenza A/B, RSV	Nasopharyngeal swabs	Hospitalized children with acute respiratory infection	Pediatric; ≤16 year	Laboratory RT-PCR*	148	34	9	1,510	1,701
Nolte et al. (29)	USA	Diagnostic comparison	Laboratory-evaluated	cobas Liat	Automated real-time RT-PCR	Influenza A/B	Respiratory specimens	Adult and pediatric inpatients and outpatients	Mixed; 1 month–81 year	FilmArray Respiratory Panel*	96	0	0	162	258
Bell et al. (30)	USA	Diagnostic accuracy study	Laboratory-evaluated	Alere i Influenza A&B	Isothermal nucleic acid amplification	Influenza A/B	Respiratory specimens in viral transport medium	Children/adolescents represented by archived specimens	Pediatric/adolescent; 10 mo–19 y	Viral culture and RT-PCR*	161	2	13	289	465
Chapin et al. (31)	USA	Diagnostic comparison	Laboratory-evaluated	Alere i	Isothermal nucleic acid amplification	Influenza A/B	Nasopharyngeal specimens	Patients with influenza-like illness	Mixed; 3 week–96 year	Xpert Flu and/or xTAG RVP*	225	22	16	30	293
Schmidt et al. (32)	Austria	Diagnostic accuracy study	Genuine near-patient	cobas Liat	Automated real-time RT-PCR	Influenza A/B	Respiratory specimens	Inpatients/outpatients with influenza-like illness or acute RTI	Mixed; median 52 y	Laboratory RT-PCR*	241	23	3	1,035	1,302

The assays detected viral RNA through platform-specific genomic targets. Across the represented systems, typical targets included the matrix (M), polymerase basic 2 (PB2), and/or polymerase acidic (PA) genes for influenza A; nonstructural (NS1/NS2) and/or M genes for influenza B; nucleocapsid (N), M, and/or fusion (F) genes for RSV; and open reading frame 1ab (ORF1ab)/RNA-dependent RNA polymerase (RdRp), envelope (E), nucleocapsid (N/N2), spike (S), and/or membrane (M) genes for SARS-CoV-2 (13). The exact target combination varied by assay platform, and complete primer and probe sequences were not always publicly reported.

Some studies reported multiple pathogen-, platform-, specimen-, or setting-specific datasets. For the primary analysis, one platform and one non-nested cohort were selected from each study according to the prespecified hierarchy. Target-specific (2 \times 2) counts within the selected platform and cohort were subsequently aggregated to form one study-level composite target-test record. The characteristics of the selected primary records are presented in Table 1. Some studies reported multiple pathogen-, platform-, specimen-, or setting-specific datasets. For the primary analysis, one platform and one non-nested cohort were selected from each study according to the prespecified hierarchy. Target-specific (2 \times 2) counts within the selected platform and cohort were subsequently aggregated to form one study-level composite target-test record. The characteristics of the selected primary records are presented in Table 1.

### 3.3 Quality assessment

The methodological quality of the included studies was evaluated using QUADAS-2 (Supplementary Table S4). In the patient-selection domain, seven studies (35.0%) were judged to have a high risk of bias, seven (35.0%) an unclear risk, and six (30.0%) a low risk. The index-test domain was judged to have an unclear risk of bias in 19 studies (95.0%) and a low risk in one study (5.0%). Sixteen studies (80.0%) had a low risk of bias in the reference-standard domain, whereas four (20.0%) had an unclear risk. In the flow-and-timing domain, 14 studies (70.0%) were judged to have a low risk of bias, three (15.0%) an unclear risk, and three (15.0%) a high risk. Applicability concerns were judged to be low across all three applicability domains in all included studies.

### 3.4 Overall diagnostic accuracy

As a broad exploratory summary of the combined evidence base, the primary analysis included 20 independent study-level composite records. The summary sensitivity was 0.971 (95% CI, 0.955–0.982), and the pooled specificity was 0.991 (95% CI, 0.980–0.996). The corresponding positive likelihood ratio was 102.8, the negative likelihood ratio was 0.029, and the diagnostic odds ratio was 3,541.

**Figure 2:**
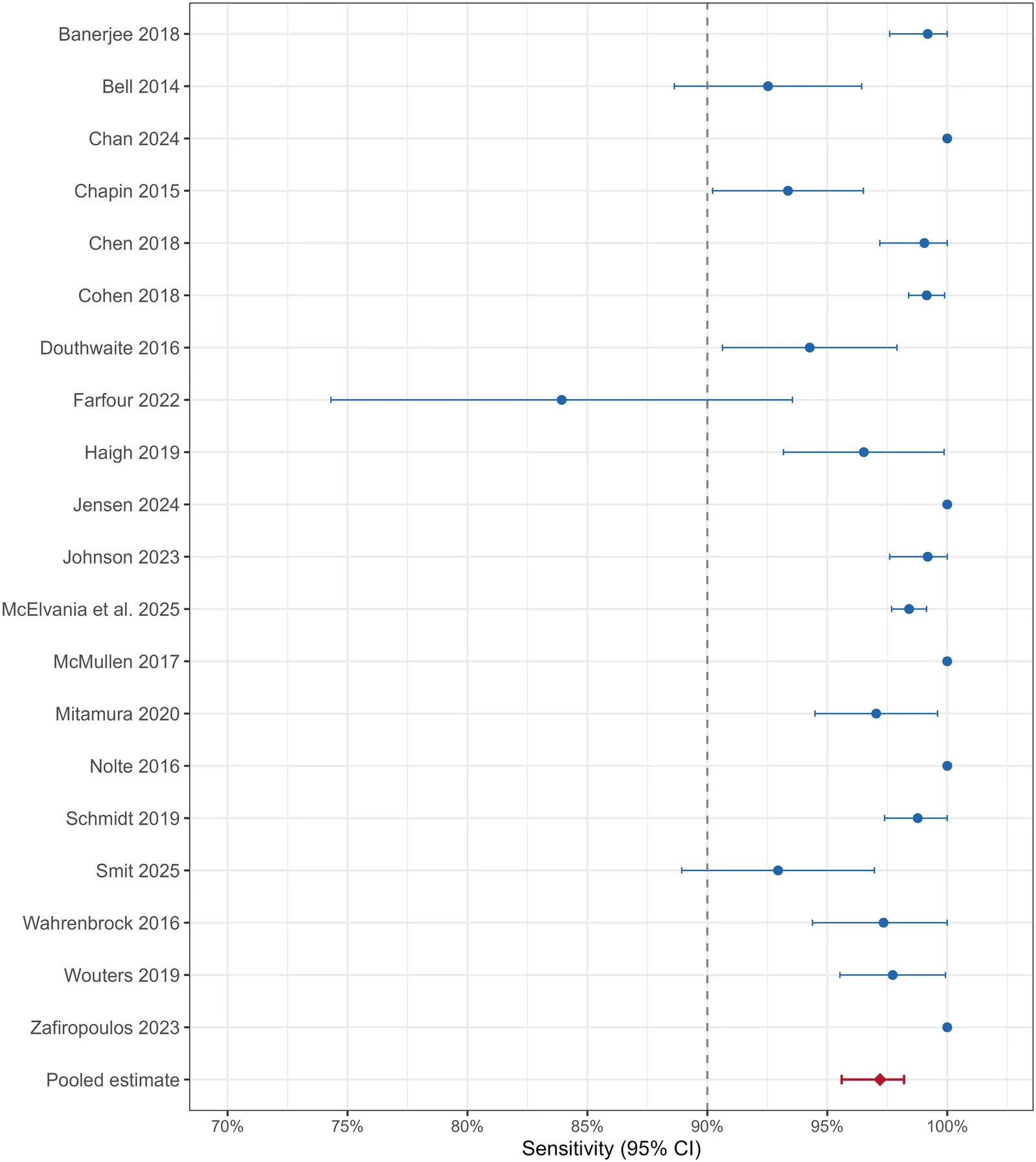
Forest plot of sensitivity.

**Figure 3:**
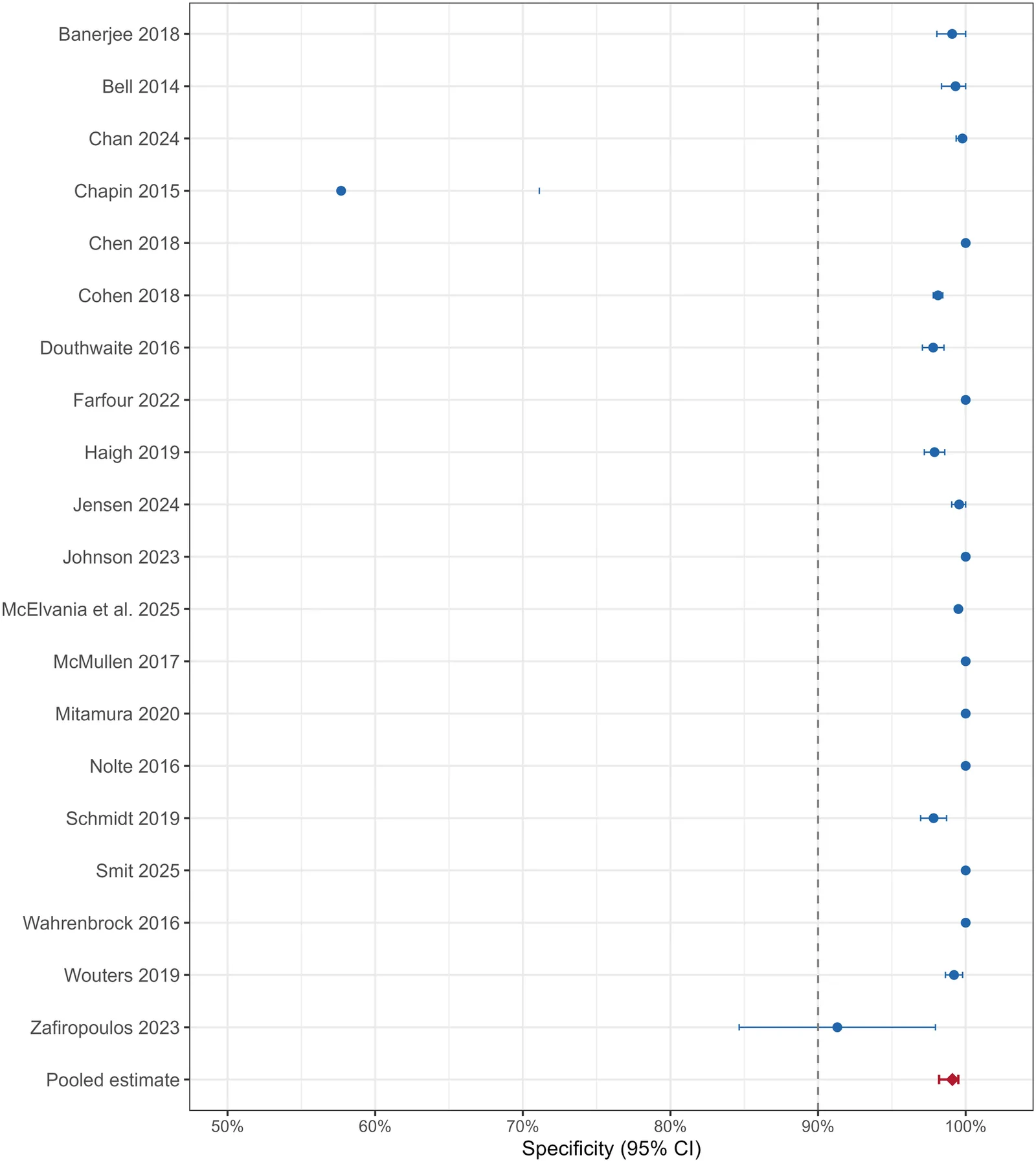
Forest plot of specificity.

The estimated between-study standard deviations on the logit scale were 0.810 for sensitivity and 1.487 for the false-positive rate. The estimated correlation between the two random effects was −0.156. Study-level sensitivity and specificity estimates are presented in Figures 2,3, respectively.

### 3.5 HSROC curve

**Figure 4:**
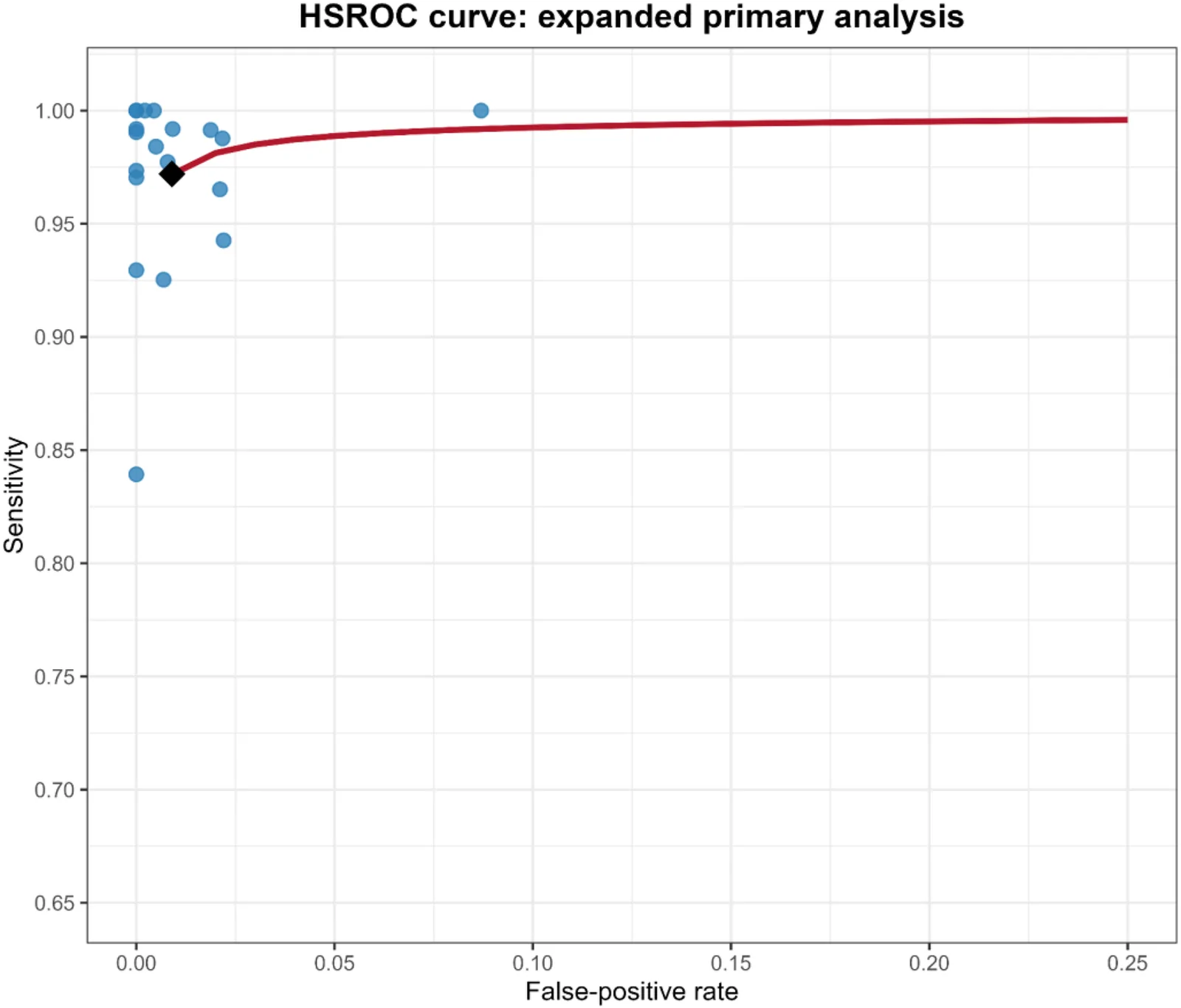
HSROC curve.

The hierarchical summary receiver operating characteristic analysis produced an area under the curve of 0.957 (Figure 4). This AUC is presented as a broad exploratory description of discrimination across the combined evidence base. The summary point was located in the upper-left region of the receiver operating characteristic space; however, study estimates showed dispersion, particularly in specificity, consistent with between-study heterogeneity in false-positive rates and the diversity of assay, specimen, population, setting, design, and reference-standard characteristics.

### 3.6 Subgroup analyses

The results of the pathogen- and setting-specific subgroup analyses are presented in Table 2. Summary sensitivity and specificity remained high across all four pathogen groups. For influenza A, the summary sensitivity was 0.970 and the summary specificity was 0.987. The corresponding estimates were 0.961 and 0.992 for influenza B, 0.955 and 0.992 for RSV, and 0.950 and 0.992 for SARS-CoV-2.

**Table 2:** Subgroup analyses according to target pathogen and operational testing setting.

Subgroup	Studies, n	Sensitivity (95% CI)	Specificity (95% CI)	PLR	NLR	DOR
Flu A	19	0.970 (0.943–0.985)	0.987 (0.974–0.994)	75.8	0.030	2,504
Flu B	18	0.961 (0.943–0.973)	0.992 (0.980–0.996)	113.7	0.039	2,883
RSV	12	0.955 (0.914–0.977)	0.992 (0.981–0.996)	115.8	0.045	2,572
SARS-CoV-2	6	0.950 (0.851–0.984)	0.992 (0.982–0.997)	122.4	0.050	2,432
Genuine near-patient	5	0.977 (0.958–0.987)	0.984 (0.974–0.991)	62.0	0.024	2,622
Laboratory-evaluated rapid integrated testing	15	0.968 (0.942–0.982)	0.993 (0.981–0.998)	144.6	0.032	4,463

The SARS-CoV-2 subgroup included only six studies and had a wider confidence interval for sensitivity than the other pathogen subgroups. Genuine near-patient studies had a summary sensitivity of 0.977 and specificity of 0.984, whereas laboratory-evaluated rapid integrated studies had a summary sensitivity of 0.968 and specificity of 0.993.

Residual clinical and methodological heterogeneity remained within each pathogen-specific subgroup because different assay platforms, specimen types, patient populations, testing settings, study designs, and reference methods were still combined.

### 3.7 Sensitivity analysis

In the leave-one-study-out sensitivity analysis, the summary sensitivity estimates ranged from 0.968 to 0.974, and the pooled specificity estimates ranged from 0.989 to 0.992. These findings indicated that no single study had a substantial influence on the overall summary estimates. The complete results of all 20 sequential omission analyses are provided in Supplementary Table S5.

Deeks' funnel-plot asymmetry test did not indicate statistically significant small-study effects [slope = 3.208, standard erro*r* = 2.810; (t = 1.142), 18 degrees of freedom; (*P* = 0.269)].

## 4 Discussion

This systematic review and meta-analysis evaluated the diagnostic accuracy of rapid integrated molecular testing for major respiratory viral infections. Pathogen-specific analyses, which were prioritized for clinical interpretation, showed summary sensitivity and specificity estimates of 0.970 and 0.987 for influenza A, 0.961 and 0.992 for influenza B, 0.955 and 0.992 for RSV, and 0.950 and 0.992 for SARS-CoV-2, respectively. Across all 20 independent study-level records, the combined sensitivity of 0.971, specificity of 0.991, and HSROC area of 0.957 should be interpreted as broad exploratory summaries of this clinically and methodologically diverse evidence base. Leave-one-study-out analyses produced only small changes in the summary estimates, indicating that the overall findings were not driven by a single study. Deeks' test did not identify statistically significant funnel-plot asymmetry. Nevertheless, these results describe diagnostic performance and should not be interpreted as direct evidence of improvements in antimicrobial prescribing, infection control, healthcare costs, length of stay, or patient outcomes.

The high summary accuracy observed in this review is broadly consistent with previous evaluations of rapid molecular assays for respiratory infections. Mojebi et al. reported that rapid molecular diagnostic testing could shorten the time to pathogen identification and facilitate earlier clinical decision-making, although the magnitude of its clinical effects varied across implementation settings (33). Evaluations of contemporary multiplex platforms have also reported strong agreement with comparator molecular assays. These include studies of Xpert Xpress CoV-2/Flu/RSV Plus in laboratory and CLIA-waived environments (24). Studies evaluating contemporary multiplex platforms, including Xpert Xpress CoV-2/Flu/RSV Plus, STANDARD M10, and BIOFIRE SPOTFIRE, have also reported excellent agreement with reference molecular assays across different respiratory viruses (34). Also, Suominen et al. concluded that a STANDARD M10 Flu/RSV/SARS-CoV-2 test accurately detected seasonal respiratory pathogens which further supported the use of multiplex molecular point-of-care testing (35). AlKharsah also demonstrated the benefits of using the Xpert Xpress CoV-2/Flu/RSV Plus cartridge in terms of turnaround time and the identification of RSV and coinfections (36). These findings support the diagnostic reliability of rapid integrated molecular testing but also indicate that assay performance should be interpreted in relation to the platform, pathogen, specimen type, patient population, and operational setting.

Several technical features may explain the favorable diagnostic performance. Unlike antigen-based tests, nucleic acid amplification assays directly detect pathogen-specific genetic material and can therefore maintain sensitivity at lower viral concentrations (10, 33). Many rapid integrated platforms use closed or near-closed cartridges that combine sample processing, amplification, detection, and automated result interpretation. This design reduces manual handling and may limit contamination and operator-dependent variation (24, 37). Multiplex systems can also detect influenza A, influenza B, RSV, and SARS-CoV-2 from a single respiratory specimen, which is particularly useful when several respiratory viruses circulate concurrently (34, 36, 38). However, analytical automation alone does not make a test a genuine point-of-care assay. Testing location, operator requirements, workflow complexity, quality-control procedures, and the time from specimen collection to clinical action must also be considered.

The distinction between genuine near-patient testing and laboratory-evaluated rapid integrated testing was therefore important in this review. Five studies evaluated genuine near-patient implementation, whereas 15 evaluated rapid integrated systems in laboratory settings. The genuine near-patient subgroup had a summary sensitivity of 0.977 and specificity of 0.984, compared with 0.968 and 0.993, respectively, in the laboratory-evaluated subgroup. These differences should not be interpreted as evidence that one setting is superior because testing setting was closely associated with platform, study design, specimen type, and patient population. In addition, the near-patient subgroup contained only five studies. The subgroup findings instead suggest that high diagnostic accuracy can be achieved in both operational contexts when appropriate procedures and quality safeguards are used.

Rapid availability of accurate results may have clinical and organizational value, but such benefits were not directly synthesized in the present diagnostic-accuracy analysis. Earlier identification of a respiratory virus may support antiviral prescribing, isolation decisions, patient cohorting, and antimicrobial stewardship. A recent systematic review and meta-analysis suggested that molecular point-of-care testing may influence antibiotic use and selected clinical outcomes in acute respiratory tract infections (39, 40). Expert recommendations have also identified potential roles for rapid molecular testing in addressing diagnostic gaps and supporting more targeted patient management (41). Nevertheless, clinical effects depend on whether results are returned early enough to change management and whether they are incorporated into defined treatment, isolation, and stewardship pathways. Rapid testing alone may not improve outcomes when clinicians do not act on the result. Separate comparative-effectiveness and health-economic studies are therefore required to determine whether diagnostic speed translates into reductions in antibiotic exposure, isolation duration, length of stay, resource utilization, or adverse patient outcomes.

Several limitations should be considered. First, although the primary analysis used one independent record per study, target-specific (2 \times 2) counts within the selected platform and cohort were aggregated into composite target-test records. The resulting denominators represented target-test pairs rather than unique patients, and correlations among pathogen results obtained from the same participants could not be modeled directly. Second, substantial clinical and methodological diversity remained across assay platforms, specimen types, patient populations, age groups, study designs, testing locations, and reference standards. Several categories contained only one or two studies, and important study characteristics overlapped substantially. Consequently, further subgroup analyses or multivariable bivariate meta-regression would have been underpowered, unstable, and vulnerable to collinearity and ecological confounding. Third, only five studies evaluated genuine near-patient implementation, limiting the precision and generalizability of the setting comparison. The SARS-CoV-2 subgroup included only six studies and consequently had a wider confidence interval for sensitivity. Fourth, several studies had a high or unclear risk of bias, particularly in patient selection and index-test reporting. Variation in reference methods and incomplete reporting of positivity thresholds or discordant-result resolution may also have influenced the estimates. Fifth, Deeks' funnel-plot asymmetry test has limited power when relatively few studies are available; therefore, the nonsignificant result does not exclude publication bias or other small-study effects. Finally, the review focused on diagnostic accuracy and did not quantitatively synthesize turnaround time, antibiotic use, antiviral initiation, isolation practices, costs, length of stay, or patient-centered outcomes.

Future studies should use prospective, multicenter designs with consecutive patient recruitment, clearly defined testing locations, standardized molecular reference methods, and transparent procedures for resolving discordant results. Investigators should report pathogen-specific (2 \times 2) data together with the number of unique participants and specimens, allowing correlations among multiple targets to be modeled appropriately. Direct comparisons of near-patient and laboratory-based implementation should evaluate the entire diagnostic pathway, including specimen collection, time to result, time to clinical action, treatment modification, infection-control decisions, resource use, and patient outcomes. Such evidence will be necessary to determine when the high diagnostic accuracy of rapid integrated molecular testing translates into meaningful clinical and economic benefit.

## 5 Conclusion

Pathogen-specific analyses suggested high diagnostic accuracy for rapid integrated molecular testing of influenza A, influenza B, RSV, and SARS-CoV-2. The combined sensitivity, specificity, and HSROC area are broad exploratory summaries. Residual clinical and methodological heterogeneity remained within each pathogen subgroup, and most studies were laboratory based. Further prospective, adequately stratified implementation studies are needed to define assay- and setting-specific performance and determine whether rapid pathogen identification improves clinical or economic outcomes.

## Data Availability

Publicly available datasets were analyzed in this study. This data can be found here: Not applicable. No public repository or accession number was used for this study. The data analyzed in this systematic review and meta-analysis were extracted from previously published studies listed in the References. All relevant source data can be found in the original published articles, and further inquiries can be directed to the corresponding author.

## References

[1] Shi T McAllister DA O'Brien KL Simoes EAF Madhi SA Gessner BD. Global, regional, and national disease burden estimates of acute lower respiratory infections due to respiratory syncytial virus in young children in 2015: a systematic review and modelling study. Lancet. (2017) 390(10098):946–58. 10.1016/S0140-6736(17)30938-8 28689664 PMC5592248

[2] Troeger CE Blacker BF Khalil IA Zimsen SRM Albertson SB Abate D. Mortality, morbidity, and hospitalisations due to influenza lower respiratory tract infections, 2017: an analysis for the Global Burden of Disease Study 2017. Lancet Respir Med. (2019) 7(1):69–89. 10.1016/S2213-2600(18)30496-X 30553848 PMC6302221

[3] Falsey AR Hennessey PA Formica MA Cox C Walsh EE. Respiratory syncytial virus infection in elderly and high-risk adults. Exp Lung Res. (2005) 31(Suppl 1):77–1759. 10.1056/NEJMoa043951 16395866

[4] Jain S Self WH Wunderink RG Fakhran S Balk R Bramley AM. Community-acquired pneumonia requiring hospitalization among U.S. Adults. N Engl J Med. (2015) 373(5):415–27. 10.1056/NEJMoa1500245 26172429 PMC4728150

[5] Caliendo AM Gilbert DN Ginocchio CC Hanson KE May L Quinn TC. Better tests, better care: improved diagnostics for infectious diseases. Clin Infect Dis. (2013) 57(Suppl 3):S139–70. 10.1093/cid/cit578 24200831 PMC3820169

[6] Merckx J Wali R Schiller I Caya C Gore GC Chartrand C. Diagnostic accuracy of novel and traditional rapid tests for influenza infection compared with reverse transcriptase polymerase chain reaction: a systematic review and meta-analysis. Ann Intern Med. (2017) 167(6):394–409. 10.7326/M17-0848 28869986

[7] Brendish NJ Malachira AK Armstrong L Houghton R Aitken S Nyimbili E. Routine molecular point-of-care testing for respiratory viruses in adults presenting to hospital with acute respiratory illness (ResPOC): a pragmatic, open-label, randomised controlled trial. Lancet Respir Med. (2017) 5(5):401–11. 10.1016/S2213-2600(17)30120-0 28392237 PMC7164815

[8] Rappo U Schuetz AN Jenkins SG Calfee DP Walsh TJ Wells MT. Impact of early detection of respiratory viruses by Multiplex PCR assay on clinical outcomes in adult patients. J Clin Microbiol. (2016) 54(8):2096–103. 10.1128/JCM.00549-16 27225406 PMC4963510

[9] Babady NE England MR Jurcic Smith KL He T Wijetunge DS Tang Y-W. Multicenter evaluation of the ePlex respiratory pathogen panel for the detection of viral and bacterial respiratory tract pathogens in nasopharyngeal swabs. J Clin Microbiol. (2018) 56(2):e01658–17. 10.1128/JCM.01658-17 29212701 PMC5786739

[10] Isles N Badman SG Ballard S Zhang B Howden BP Guy R. Analytical sensitivity and specificity of the Cepheid Xpert Xpress SARS-CoV-2/Flu/RSV assay. Pathology. (2022) 54(1):120–2. 10.1016/j.pathol.2021.09.002 34776245 PMC8531023

[11] Page MJ McKenzie JE Bossuyt PM Boutron I Hoffmann TC Mulrow CD. The PRISMA 2020 statement: an updated guideline for reporting systematic reviews. BMJ (Clinical Research ed.). (2021) 372:n71. 10.1136/bmj.n71 33782057 PMC8005924

[12] Whiting PF Rutjes AWS Westwood ME Mallett S Deeks JJ Reitsma JB. QUADAS-2: a revised tool for the quality assessment of diagnostic accuracy studies. Ann Intern Med. (2011) 155(8):529–36. 10.7326/0003-4819-155-8-201110180-00009 22007046

[13] Smit PW Man P Brand H Breijer S Wijk M Meijer A. Comparative evaluation of five rapid PCR platforms for respiratory virus detection. PLoS One. (2025) 20(12):e0338716. 10.1371/journal.pone.0338716 41379874 PMC12697972

[14] Haigh J Cutino-Moguel M-T Wilks M Welch CA Melzer M. A service evaluation of simultaneous near-patient testing for influenza, respiratory syncytial virus, Clostridium difficile and norovirus in a UK district general hospital. J Hosp Infect. (2019) 103(4):441–6. 10.1016/j.jhin.2019.08.022 31494128

[15] Chen JH Lam HY Yip CC Cheng VC Chan JF Leung TH. Evaluation of the molecular Xpert Xpress Flu/RSV assay vs. Alere i Influenza A & B assay for rapid detection of influenza viruses. Diagn Microbiol Infect Dis. (2018) 90(3):177–80. 10.1016/j.diagmicrobio.2017.11.010 29262988

[16] Mitamura K Shimizu H Yamazaki M Ichikawa M Abe T Yasumi Y. Clinical evaluation of ID NOW influenza A & B 2, a rapid influenza virus detection kit using isothermal nucleic acid amplification technology - A comparison with currently available tests. J Infect Chemother. (2020) 26(2):216–21. 10.1016/j.jiac.2019.08.015 31558351

[17] Jensen CB Schneider UV Madsen TV Nielsen XC Ma CMG Severinsen JK. Evaluation of the analytical and clinical performance of two RT-PCR based point-of-care tests; Cepheid Xpert® Xpress CoV-2/Flu/RSV plus and SD BioSensor STANDARD™ M10 Flu/RSV/SARS-CoV-2. J Clin Virol. (2024) 172:105674. 10.1016/j.jcv.2024.105674 38643722

[18] Wouters Y Keyaerts E Rector A Van Even E Vissers S Koletzki D. Comparison of the Idylla™ respiratory (IFV-RSV) panel with the GeneXpert Xpert® Flu/RSV assay: a retrospective study with nasopharyngeal and midturbinate samples. Diagn Microbiol Infect Dis. (2019) 94(1):33–7. 10.1016/j.diagmicrobio.2018.11.022 30638655

[19] Zafiropoulos A Dermitzaki A Malliarakis N Stamataki M Ergazaki M Xenaki E. Comparison of two point-of-care respiratory panels for the detection of influenza A/B virus. Infect Dis (London, England). (2023) 55(7):509–13. 10.1080/23744235.2023.2214611 37198922

[20] Johnson G Gregorchuk BSJ Zubrzycki A Kolsun K Meyers AFA Sandstrom PA. Clinical evaluation of the GeneXpert(®) Xpert(®) Xpress SARS-CoV-2/Flu/RSV PLUS combination test. Can J Microbiol. (2023) 69(3):146–50. 10.1139/cjm-2022-0204 36657122

[21] Banerjee D Kanwar N Hassan F Essmyer C Selvarangan R. Comparison of six sample-to-answer influenza A/B and respiratory syncytial virus nucleic acid amplification assays using respiratory specimens from children. J Clin Microbiol. (2018) 56(11):e00930–18. 10.1128/JCM.00930-18 30185508 PMC6204686

[22] Cohen DM Kline J May LS Harnett GE Gibson J Liang SY. Accurate PCR detection of influenza A/B and respiratory syncytial viruses by use of Cepheid Xpert Flu + RSV Xpress assay in point-of-care settings: comparison to prodesse ProFlu. J Clin Microbiol. (2018) 56(2):e01237–17. 10.1128/JCM.01237-17 29142048 PMC5786727

[23] Farfour E Yung T Baudoin R Vasse M. Evaluation of four fully integrated molecular assays for the detection of respiratory viruses during the Co-circulation of SARS-CoV-2, influenza and RSV. J Clin Med. (2022) 11(14):3942. 10.3390/jcm11143942 35887705 PMC9317686

[24] McElvania E Rao D Greninger AL Harnett G Larcena A Patel A. Evaluation of Cepheid Xpert Xpress CoV-2/Flu/RSV plus for nasal and nasopharyngeal specimens tested in CLIA-accredited and CLIA-waived settings. J Clin Virol. (2025) 180:105851. 10.1016/j.jcv.2025.105851 40818320

[25] Chan W-S Ho CW-Y Chan T-C Hung J To M-Y Leung S-M. Clinical evaluation of the BIOFIRE SPOTFIRE respiratory panel. Viruses. (2024) 16(4):600. 10.3390/v16040600 38675941 PMC11055108

[26] Chen JHK Lam H-Y Yip CCY Wong SCY Chan JFW Ma ESK. Clinical evaluation of the new high-throughput luminex NxTAG respiratory pathogen panel assay for multiplex respiratory pathogen detection. J Clin Microbiol. (2016) 54(7):1820–5. 10.1128/JCM.00517-16 27122380 PMC4922091

[27] Wahrenbrock MG Matushek S Boonlayangoor S Tesic V Beavis KG Charnot-Katsikas A. Comparison of cepheid Xpert flu/RSV XC and BioFire FilmArray for detection of influenza A, influenza B, and respiratory syncytial virus. J Clin Microbiol. (2016) 54(7):1902–3. 10.1128/JCM.00084-16 27098956 PMC4922080

[28] Douthwaite ST Walker C Adams EJ Mak C Vecino Ortiz A Martinez-Alier N. Performance of a novel point-of-care molecular assay for detection of influenza A and B viruses and respiratory syncytial virus (Enigma MiniLab) in children with acute respiratory infection. J Clin Microbiol. (2016) 54(1):212–5. 10.1128/JCM.02887-15 26560540 PMC4702736

[29] Nolte FS Gauld L Barrett SB. Direct comparison of Alere i and cobas liat influenza A and B tests for rapid detection of influenza virus infection. J Clin Microbiol. (2016) 54(11):2763–6. 10.1128/JCM.01586-16 27582513 PMC5078555

[30] Bell JJ Selvarangan R. Evaluation of the alere I influenza A&B nucleic acid amplification test by use of respiratory specimens collected in viral transport medium. J Clin Microbiol. (2014) 52(11):3992–5. 10.1128/JCM.01639-14 25210070 PMC4313236

[31] Chapin KC Flores-Cortez EJ. Performance of the molecular Alere i influenza A&B test compared to that of the xpert flu A/B assay. J Clin Microbiol. (2015) 53(2):706–9. 10.1128/JCM.02783-14 25502527 PMC4298524

[32] Schmidt RLJ Simon A Popow-Kraupp T Laggner A Haslacher H Fritzer-Szekeres M. A novel PCR-based point-of-care method facilitates rapid, efficient, and sensitive diagnosis of influenza virus infection. Clin Microbiol Infect. (2019) 25(8):1032–7. 10.1016/j.cmi.2018.12.017 30583060

[33] Mojebi A Wu P Keeping S Hale B Chase JG Beaubrun A. Clinical impact of rapid molecular diagnostic tests in patients presenting with viral respiratory symptoms: a systematic literature review. PLoS One. (2024) 19(6):e0303560. 10.1371/journal.pone.0303560 38870136 PMC11175541

[34] Chan W-S Wong K-P Yau S-K Wong C-Y Chan T-C Hung J. Clinical evaluation of Xpert Xpress CoV-2/flu/RSV plus and Alinity m Resp-4-plex assay. Diagnostics (Basel). (2024) 14(7):683. 10.3390/diagnostics14070683 38611596 PMC11012021

[35] Suominen J Loginov R Kallio-Kokko H. Comparative evaluation of STANDARD™ M10 flu/RSV/SARS-CoV-2 and savanna® respiratory viral panel-4 assays for the rapid molecular diagnosis of influenza A/B virus, respiratory syncytial virus and SARS-CoV-2. J Clin Virol. (2025) 179:105827. 10.1016/j.jcv.2025.105827 40513219

[36] Alkharsah KR. Utility of the Xpert Xpress CoV-2/flu/RSV plus kit: a glance at RSV infection in adults and coinfection rate. Saudi J Med Med Sci. (2024) 12(2):182–7. 10.4103/sjmms.sjmms_376_23 38764565 PMC11098272

[37] Sedik S Wolfgruber S Hoenigl M Kriegl L. Diagnosing fungal infections in clinical practice: a narrative review. Expert Rev Anti-Infect Ther. (2024) 22(11):935–49. 10.1080/14787210.2024.2403017 39268795

[38] Domnich A Garzillo G Paolozzi V Ricucci V Orsi A Icardi G. Comparative diagnostic performance of the 36-Minute STANDARD M10 fast assay for molecular diagnosis of SARS-CoV-2, influenza A and B, and respiratory syncytial virus in near-patient settings. Infect Dis Ther. (2026) 15(2):591–604. 10.1007/s40121-025-01284-2 41483387 PMC12855711

[39] Li Q Zhou Q Fan J Feng X Lai H Chen Y. Impact of molecular point-of-care testing for respiratory pathogens on antibiotic use and clinical outcomes in acute respiratory tract infections: a systematic review and meta-analysis. eClinicalMed. (2026) 92:103799. 10.1016/j.eclinm.2026.103799 PMC12925124 41732193

[40] Vos LM Bruning AHL Reitsma JB Schuurman R Riezebos-Brilman A Hoepelman AIM. Rapid molecular tests for influenza, respiratory syncytial virus, and other respiratory viruses: a systematic review of diagnostic accuracy and clinical impact studies. Clin Infect Dis. (2019) 69(7):1243–53. 10.1093/cid/ciz056 30689772 PMC7108200

[41] Dhaini L Verma R Gadir MA Singh H Farghaly M Abdelmutalib T. Recommendations on rapid diagnostic point-of-care molecular tests for respiratory infections in the United Arab Emirates. Open Respir Med J. (2024) 18:e18743064319029. 10.2174/0118743064319029240815074449 39872239 PMC11770827

