# Supplementary material for: Diagnostic accuracy of rapid integrated molecular testing for major respiratory viral infections: a systematic review and meta-analysis
Source: Front Med (Lausanne). 2026 Sep 11;13:1909562. doi: 10.3389/fmed.2026.1909562 (PMC13612272; doi:10.3389/fmed.2026.1909562)
Supplement: Supplementary file 1 [file Datasheet1.docx]

**Diagnostic Accuracy of Rapid Integrated Molecular Testing for Major Respiratory Viral Infections: A Systematic Review and Meta-analysis**

**Supplementary File S1. Complete database-specific search strategies**

Final search date: June 2026. Databases were searched from inception. Language restriction: non.

**Cochrane Library search strategy**

("point-of-care" OR "near-patient" OR POCT OR "rapid molecular" OR GeneXpert OR Xpert OR FilmArray OR BioFire OR "ID NOW" OR "cobas Liat" OR "Alere i" OR ePlex) AND (influenza OR flu OR RSV OR "respiratory syncytial virus" OR SARS-CoV-2 OR COVID-19 OR "respiratory virus")

**Records retrieved: 105**

**Embase search strategy**

('point of care testing'/exp OR 'point of care':ti,ab,kw OR 'near patient':ti,ab,kw OR poct:ti,ab,kw OR 'rapid molecular':ti,ab,kw OR genexpert:ti,ab,kw OR xpert:ti,ab,kw OR filmarray:ti,ab,kw OR biofire:ti,ab,kw OR 'id now':ti,ab,kw OR 'cobas liat':ti,ab,kw OR 'alere i':ti,ab,kw OR eplex:ti,ab,kw) AND ('influenza'/exp OR influenza:ti,ab,kw OR flu:ti,ab,kw OR 'respiratory syncytial virus infection'/exp OR rsv:ti,ab,kw OR 'respiratory syncytial virus':ti,ab,kw OR 'sars-related coronavirus'/exp OR 'sars-cov-2':ti,ab,kw OR 'covid-19':ti,ab,kw OR 'respiratory virus':ti,ab,kw)

**Records retrieved: 784**

**PubMed search strategy**

("Point-of-Care Testing"[Mesh] OR point-of-care[tiab] OR near-patient[tiab] OR POCT[tiab] OR rapid molecular[tiab] OR molecular point-of-care[tiab] OR GeneXpert[tiab] OR Xpert[tiab] OR FilmArray[tiab] OR BioFire[tiab] OR "ID NOW"[tiab] OR "cobas Liat"[tiab] OR "Alere i"[tiab] OR ePlex[tiab]) AND ("Influenza, Human"[Mesh] OR influenza[tiab] OR flu[tiab] OR "Respiratory Syncytial Viruses"[Mesh] OR RSV[tiab] OR "respiratory syncytial virus"[tiab] OR SARS-CoV-2[tiab] OR COVID-19[tiab] OR respiratory virus*[tiab])

**Records retrieved: 448**

**Supplementary Table S1. Operational eligibility definition**

| Category | Location | Workflow | Turnaround | Eligibility |
| --- | --- | --- | --- | --- |
| Genuine near-patient rapid integrated | Clinic, ED, ward, bedside, or waived site | Closed/near-closed sample-to-answer; minimal manipulation | ≤120 min | Included |
| Laboratory-evaluated rapid integrated | Local, satellite, or central laboratory | Integrated sample-to-answer platform | ≤120 min | Included |
| Conventional centralized/batch molecular | Central laboratory | Off-instrument extraction, batch amplification, or multi-step workflow | Not sufficient | Excluded |

**Supplementary Table S2. Characteristics and reference methods of the included studies**

| Study | Country | Design/population | Testing setting | Specimen | Selected index test | Reference method and discordant-result handling |
| --- | --- | --- | --- | --- | --- | --- |
| Banerjee 2018 | USA | Comparative accuracy; enriched pediatric respiratory specimens | Laboratory | Pediatric respiratory specimens | cobas Liat | CDC influenza rRT-PCR and CDC RSV rRT-PCR; reference testing used for discrepant analysis |
| Bell 2014 | USA | Diagnostic accuracy; historically characterized specimens | Laboratory | Midturbinate swabs and NP wash/aspirates in VTM | Alere i Influenza A&B | R-Mix shell-vial viral culture and Prodesse ProFlu+ real-time RT-PCR |
| Chan 2024 | Hong Kong, China | Clinical laboratory evaluation; archived URT specimens | Laboratory | NPS and other URT specimens | BIOFIRE SPOTFIRE Respiratory Panel | BIOFIRE FilmArray RP2.1plus; discrepant results verified with target-specific alternative molecular methods |
| Chapin 2015 | USA | Retrospective comparison; residual frozen specimens | Laboratory | NP swabs in UTM | Alere i Influenza A&B | Xpert Flu A/B; xTAG Respiratory Viral Panel used for discrepant resolution |
| Chen 2018 | Hong Kong, China | Diagnostic comparison of 134 clinical specimens | Laboratory | Nasopharyngeal aspirates/swabs | Xpert Xpress Flu/RSV | In-house multiplex influenza A/B real-time RT-PCR LDT using Path-ID Multiplex One-Step RT-PCR |
| Cohen 2018 | USA | Multicenter diagnostic accuracy; prospective plus preselected frozen specimens | Predominantly CLIA-waived | NP swabs in UTM | Xpert Flu/RSV Xpress | FDA-cleared Prodesse ProFlu+ real-time RT-PCR; discordant results resolved by bidirectional sequencing with independent primers |
| Douthwaite 2016 | UK | Service evaluation in children with acute respiratory infection | Ward/near-patient | Pediatric respiratory specimens | Enigma MiniLab FluAB-RSV | Luminex xTAG Respiratory Viral Panel; discrepant results resolved with Xpert Flu/RSV XC and/or additional PCR |
| Farfour 2022 | France | Prospective emergency-department diagnostic comparison | Laboratory | Fresh NPS in UTM | Idylla SARS-CoV-2/Flu/RSV | Alinity m RESP-4-Plex multiplex RT-PCR; reference assay repeated for discrepant results |
| Brendish 2019 | UK | Prospective service evaluation | Local near-patient | Respiratory specimens | GeneXpert Xpert Flu/RSV | Centralized QIAsymphony-based multiplex PCR, including AusDiagnostics High-Plex Upper Respiratory Pathogens MT-PCR and an in-house influenza triplex PCR; third-party testing for selected discordances |
| Jensen 2024 | Denmark | Analytical and clinical comparison using 492 clinical specimens | Laboratory | Respiratory specimens | Xpert Xpress CoV-2/Flu/RSV Plus | Standard-of-care RT-PCR; QIAstat-Dx Respiratory SARS-CoV-2 Panel used for discrepant testing |
| Johnson 2023 | Canada | Clinical/analytical comparison using residual characterized specimens | Laboratory | Nasal and NP swab material in UTM | Xpert Xpress CoV-2/Flu/RSV Plus | Previous Xpert Xpress SARS-CoV-2/Flu/RSV combination assay; selected specimens were also characterized by laboratory-developed RT-qPCR |
| McElvania 2025 | USA | Prospective multicenter diagnostic accuracy at 33 healthcare sites | CLIA-waived/nonlaboratory cohort | Nasal and NP swabs | Xpert Xpress CoV-2/Flu/RSV Plus | BIOFIRE RP2.1 for SARS-CoV-2 and Hologic Panther Fusion Flu A/B/RSV for influenza/RSV; target-specific discrepant testing |
| McMullen 2017 | USA | Prospective diagnostic comparison | Laboratory | Respiratory specimens | Luminex ARIES Flu A/B & RSV | Cepheid Xpert Flu/RSV XC molecular assay |
| Mitamura 2020 | Japan | Prospective clinical evaluation in patients with influenza-like illness | Laboratory | Nasopharyngeal specimens | ID NOW Influenza A&B 2 | Real-time RT-PCR reference method; viral culture and LAMP were additional comparators |
| Nolte 2016 | USA | Direct comparison using selected residual specimens | Laboratory | Respiratory specimens | cobas Liat Influenza A/B | BIOFIRE FilmArray Respiratory Panel; selected discordances tested by quantitative influenza A real-time RT-PCR |
| Schmidt 2019 | Austria | Prospective single-center method comparison | Emergency department/on-site | Nasal and NP swabs | cobas Liat | Central-laboratory Xpert Flu assay; common-arm specimens additionally tested with Xpert Xpress Flu/RSV |
| Smit 2025 | Netherlands | Prospective comparative evaluation of rapid PCR platforms | Laboratory | Nasopharyngeal samples | STANDARD M10 (1-hour configuration) | GeneXpert Xpert Xpress CoV-2/Flu/RSV used as the reference method |
| Wahrenbrock 2016 | USA | Method-comparison study | Laboratory | NP swabs | Xpert Flu/RSV XC | BIOFIRE FilmArray Respiratory Panel; discordant results underwent additional molecular testing |
| Wouters 2019 | Belgium | Retrospective comparison of residual samples | Laboratory | Nasopharyngeal and midturbinate swabs | Idylla Respiratory IFV-RSV Panel | GeneXpert Xpert Flu/RSV as imperfect reference; discordances resolved by in-house influenza/RSV RT-PCR at UZ Leuven/KU Leuven |
| Zafiropoulos 2023 | Greece | Diagnostic comparison in patients with acute respiratory infection | Laboratory | Respiratory swabs | BIOFIRE FilmArray RP2plus | Alere i Influenza A&B molecular assay; no additional third-method resolution reported |

*Abbreviations: LAMP, loop-mediated isothermal amplification; LDT, laboratory-developed test; NP, nasopharyngeal; NPS, nasopharyngeal swab; rRT-PCR, real-time reverse-transcription polymerase chain reaction; RSV, respiratory syncytial virus; UTM, universal transport medium; URT, upper respiratory tract; VTM, viral transport medium.*

**Supplementary Table S3. Study-level primary diagnostic dataset and dependence handling**

| Study | Selected platform | Targets | Setting | TP | FP | FN | TN | Target-test total | Selection/dependence handling |
| --- | --- | --- | --- | --- | --- | --- | --- | --- | --- |
| Banerjee 2018 | cobas Liat | Flu A; Flu B | Laboratory-evaluated | 122 | 3 | 1 | 324 | 450 | Selected platform; target rows aggregated |
| Bell 2014 | Alere i | Flu A; Flu B | Laboratory-evaluated | 161 | 2 | 13 | 289 | 465 | Target rows aggregated |
| Chan 2024 | BIOFIRE SPOTFIRE RP | Flu A; Flu B; RSV; SARS-CoV-2 | Laboratory-evaluated | 73 | 1 | 0 | 458 | 532 | Target rows aggregated |
| Chapin 2015 | Alere i | Flu A; Flu B | Laboratory-evaluated | 225 | 22 | 16 | 30 | 293 | Target rows aggregated |
| Chen 2018 | Xpert Xpress | Flu A; Flu B | Laboratory-evaluated | 104 | 0 | 1 | 163 | 268 | Selected platform; target rows aggregated |
| Cohen 2018 | Xpert Flu/RSV Xpress | Flu A; Flu B; RSV | Genuine near-patient | 578 | 126 | 5 | 6596 | 7305 | Overall cohort retained; target rows aggregated |
| Douthwaite 2016 | Enigma MiniLab | Flu A; Flu B; RSV | Genuine near-patient | 148 | 34 | 9 | 1510 | 1701 | Ward cohort; target rows aggregated |
| Farfour 2022 | Idylla SARS-CoV-2/Flu/RSV | Flu A; RSV; SARS-CoV-2 | Laboratory-evaluated | 47 | 0 | 9 | 598 | 654 | Selected platform; target rows aggregated |
| Brendish 2019 | GeneXpert Xpert Flu/RSV | Flu A; Flu B; RSV | Genuine near-patient | 111 | 35 | 4 | 1623 | 1773 | Local near-patient cohort; target rows aggregated |
| Jensen 2024 | Xpert Xpress Plus | Flu A; Flu B; RSV; SARS-CoV-2 | Laboratory-evaluated | 320 | 3 | 0 | 673 | 996 | Xpert selected; target rows aggregated |
| Johnson 2023 | Xpert Xpress Plus | Flu A; Flu B; RSV; SARS-CoV-2 | Laboratory-evaluated | 122 | 0 | 1 | 517 | 640 | Target rows aggregated |
| McElvania 2025 | Xpert Xpress Plus | Flu A; Flu B; RSV; SARS-CoV-2 | Genuine near-patient | 1114 | 67 | 18 | 13442 | 14641 | CLIA-waived/nonlaboratory cohort; target rows aggregated |
| McMullen 2017 | Luminex ARIES | Flu A; Flu B; RSV | Laboratory-evaluated | 53 | 0 | 0 | 376 | 429 | Selected platform; target rows aggregated |
| Mitamura 2020 | ID NOW Influenza A&B 2 | Flu A; Flu B | Laboratory-evaluated | 164 | 0 | 5 | 373 | 542 | Target rows aggregated |
| Nolte 2016 | cobas Liat | Flu A; Flu B | Laboratory-evaluated | 96 | 0 | 0 | 162 | 258 | cobas Liat selected; target rows aggregated |
| Schmidt 2019 | cobas Liat | Flu A; Flu B | Genuine near-patient | 241 | 23 | 3 | 1035 | 1302 | On-site cohort; target rows aggregated |
| Smit 2025 | STANDARD M10 (1 hour) | Flu A; Flu B; RSV; SARS-CoV-2 | Laboratory-evaluated | 145 | 0 | 11 | 456 | 612 | M10 1-hour configuration selected |
| Wahrenbrock 2016 | Xpert Flu/RSV XC | Flu A; Flu B; RSV | Laboratory-evaluated | 110 | 0 | 3 | 274 | 387 | Target rows aggregated |
| Wouters 2019 | Idylla Respiratory Panel | Flu A; Flu B; RSV | Laboratory-evaluated | 172 | 7 | 4 | 879 | 1062 | NPS cohort selected; target rows aggregated |
| Zafiropoulos 2023 | BIOFIRE RP2plus | Influenza A/B combined | Laboratory-evaluated | 28 | 6 | 0 | 63 | 97 | Single combined influenza record |

*Statistical note: Target-specific 2 × 2 counts were aggregated only within the prespecified platform and non-nested cohort selected from each study. The resulting denominators represent target-test pairs rather than unique patients or specimens. Multiple target-specific results from the same study were not treated as statistically independent in the primary analysis.*

*Abbreviations: FN, false negative; FP, false positive; RSV, respiratory syncytial virus; TN, true negative; TP, true positive.*

**Supplementary Table S4. Study-level QUADAS-2 judgments**

| Study | Patient selection | Index test | Reference standard | Flow and timing | Applicability: patient | Applicability: index | Applicability: reference |
| --- | --- | --- | --- | --- | --- | --- | --- |
| Banerjee 2018 | High | Unclear | Low | Low | Low | Low | Low |
| Bell 2014 | High | Unclear | Low | Unclear | Low | Low | Low |
| Chan 2024 | High | Low | Unclear | Low | Low | Low | Low |
| Chapin 2015 | High | Unclear | Low | High | Low | Low | Low |
| Chen 2018 | Unclear | Unclear | Low | Low | Low | Low | Low |
| Cohen 2018 | Unclear | Unclear | Low | High | Low | Low | Low |
| Douthwaite 2016 | Low | Unclear | Low | Low | Low | Low | Low |
| Farfour 2022 | Low | Unclear | Low | High | Low | Low | Low |
| Brendish 2019 | Unclear | Unclear | Low | Unclear | Low | Low | Low |
| Jensen 2024 | Unclear | Unclear | Low | Low | Low | Low | Low |
| Johnson 2023 | Unclear | Unclear | Low | Low | Low | Low | Low |
| McElvania 2025 | Low | Unclear | Low | Low | Low | Low | Low |
| McMullen 2017 | Unclear | Unclear | Low | Unclear | Low | Low | Low |
| Mitamura 2020 | Low | Unclear | Low | Low | Low | Low | Low |
| Nolte 2016 | High | Unclear | Unclear | Low | Low | Low | Low |
| Schmidt 2019 | Low | Unclear | Low | Low | Low | Low | Low |
| Smit 2025 | Low | Unclear | Low | Low | Low | Low | Low |
| Wahrenbrock 2016 | High | Unclear | Unclear | Low | Low | Low | Low |
| Wouters 2019 | High | Unclear | Unclear | Low | Low | Low | Low |
| Zafiropoulos 2023 | Unclear | Unclear | Low | Low | Low | Low | Low |

*Low, low risk of bias or low applicability concern; High, high risk of bias or high applicability concern; Unclear, insufficient information for judgment.*

**Supplementary Table S5. Leave-one-study-out sensitivity analysis**

| Omitted study | Sensitivity | Specificity | PLR | NLR | DOR |
| --- | --- | --- | --- | --- | --- |
| Banerjee 2018 | 0.970209 | 0.990797 | 105.425 | 0.030068 | 3506.3 |
| Bell 2014 | 0.973381 | 0.990601 | 103.562 | 0.026872 | 3853.9 |
| Chan 2024 | 0.970270 | 0.990095 | 97.957 | 0.030027 | 3262.2 |
| Chapin 2015 | 0.972861 | 0.991698 | 117.189 | 0.027366 | 4282.3 |
| Chen 2018 | 0.970427 | 0.990217 | 99.191 | 0.029865 | 3321.3 |
| Cohen 2018 | 0.968424 | 0.991118 | 109.035 | 0.031859 | 3422.4 |
| Douthwaite 2016 | 0.972904 | 0.991270 | 111.445 | 0.027335 | 4077.1 |
| Farfour 2022 | 0.973836 | 0.989481 | 92.575 | 0.026442 | 3501.0 |
| Brendish 2019 | 0.972091 | 0.991230 | 110.841 | 0.028155 | 3936.7 |
| Jensen 2024 | 0.969129 | 0.990377 | 100.714 | 0.031171 | 3231.0 |
| Johnson 2023 | 0.970140 | 0.989720 | 94.372 | 0.030171 | 3128.0 |
| McElvania 2025 | 0.970029 | 0.990330 | 100.309 | 0.030263 | 3314.6 |
| McMullen 2017 | 0.970491 | 0.989853 | 95.646 | 0.029812 | 3208.3 |
| Mitamura 2020 | 0.971929 | 0.989832 | 95.585 | 0.028359 | 3370.5 |
| Nolte 2016 | 0.970085 | 0.990220 | 99.187 | 0.030211 | 3283.1 |
| Schmidt 2019 | 0.970077 | 0.991209 | 110.344 | 0.030188 | 3655.2 |
| Smit 2025 | 0.973453 | 0.989685 | 94.368 | 0.026824 | 3518.1 |
| Wahrenbrock 2016 | 0.971732 | 0.989979 | 96.972 | 0.028554 | 3396.1 |
| Wouters 2019 | 0.971343 | 0.990667 | 104.074 | 0.028927 | 3597.8 |
| Zafiropoulos 2023 | 0.971282 | 0.991707 | 117.118 | 0.028958 | 4044.4 |
